# The effect of two formulations of carbon enterosorbents on oxidative stress indexes and molecular conformation of serum albumin in experimental animals exposed to CCl_4_

**DOI:** 10.1016/j.heliyon.2019.e03126

**Published:** 2020-01-06

**Authors:** Veronika Sarnatskaya, Victor Mikhailenko, Igor Prokopenko, Bogdan I. Gerashchenko, Oksana Shevchuk, Larysa Yushko, Alexei Glavin, Lyudmila Makovetska, Larysa Sakhno, Oleksii Sydorenko, Oleksandr Kozynchenko, Vladimir Nikolaev

**Affiliations:** a R.E. Kavetsky Institute of Experimental Pathology, Oncology and Radiobiology (IEPOR), NAS of Ukraine, Kyiv, Ukraine; b I. Horbachevsky Ternopil State Medical University, Ternopil, Ukraine; c ImmutriX Therapeutics Inc., Rapid City, SD, USA

**Keywords:** Pharmaceutical chemistry, Toxicology, Carbonic enterosorbents, Oxidative stress, Antioxidants, Liver failure, Serum albumin, Protein conformation, Differential scanning calorimetry, Rats

## Abstract

The liver failure means inability to perform its normal synthetic, biotransformation and excretory functions. The disturbance of metabolic processes leads to the development of "metabolic endogenous intoxication" resulting in oxidative stress. Oxidative stress initiates the processes of oxidation of amino acid residues of blood plasma proteins causing the changes in their structure and functions. The effect of administration of highly activated porous carbonic enterosorbents on oxidative stress manifestations and molecular conformation of serum albumin in blood of experimental animals with acute liver failure induced by carbon tetrachloride (CCl_4_) needs to be investigated. Two forms of activated carbonic enterosorbents such as AC1 (primary beads with the range of diameters of 125–250 μm) and AC2 (secondary granules prepared from micronized AC1 having the mean particle size of ~1 μm) derived from phenol-formaldehyde resin were used in rat model with CCl_4_ intoxication. The total level of reactive oxygen species (ROS) in blood plasma, the activity of catalase (CAT) in blood hemolysates; the content of reduced glutathione (GSH) in liver homogenates, and the level of oxidative modification of proteins (OMP) such as aldehyde-dinitrophenylhydrazone (A-DNPH) and ketone-dinitrophenylhydrazone (K-DNPH) derivatives in blood plasma and liver homogenates were determined. In addition, the level of pro/antioxidant ratio in blood hemolysates and the content of lipid peroxidation product - malondialdehyde (MDA), in blood plasma and liver were determined. Melting thermograms of blood plasma proteins (BPP) and molecular conformation changes of serum albumin were analyzed by biophysical methods (differential scanning microcalorimetry and spectrofluorimetry). The extent of CCl_4_-induced oxidative damage in blood and liver of experimental animals was shown to be less expressed for AC1 in comparison with AC2 enterosorbent. However, AC2 used in the form of secondary granules positively influenced some biophysical properties of albumin molecule (temperature of melting, shape of melting endotherm and intrinsic fluorescence) after rats exposure to CCl_4_. In general, administration of both AC1 and AC2 led to the reduction of oxidative stress manifestations and partial restoration of native molecular conformation of serum albumin. These observations are promising in terms of achieving recovery of detoxification potential of organism after severe liver injury.

## 1 Introduction

Carbon tetrachloride (CCl_4_) is widely used in *in vivo* liver injury models, and the damage induced by CCl_4_ is comparable to that observed with viral hepatitis. The main advantage of studies with CCl_4_ is in convenience of using the animal models with the evidence of achievement of fibrosis and cirrhosis as well as the study of mechanisms associated with induction of hepatotoxic states (fatty degeneration, fibrosis, hepatocellular death, and carcinogenicity). Typically, CCl_4_ is transformed into a toxic trichloromethyl radical, CCl_3_^‾•^ by CYP2E1, an enzyme expressed in perivenular hepatocytes [1]. This radical can bind to various cellular molecules impairing crucial processes of a cell, such as lipids metabolism, with the potential outcome of fatty degeneration (steatosis). Adduct formation in the result of reaction of CCl_3_^‾•^ with DNA is thought to function as initiator of hepatic cancer. By reacting with oxygen this radical transforms into the highly reactive trichloromethylperoxy radical CCl_3_OO^‾•^ that initiates the chain reaction of lipid peroxidation and damages polyunsaturated fatty acids, in particular those associated with phospholipids. These effects can result in the altered permeability of cells membranes, mitochondria [2], and endoplasmic reticulum, causing deregulation of calcium sequestration and homeostasis, which are indispensable for the normal cell functions [3]. Thus, free radicals including reactive oxygen species (ROS) related to oxidative stress can play a pivotal role in the liver injury induced by CCl_4_. The oxidative stress caused due to CCl_4_-induced free radical production is considered to be one of the main mechanism by which the hepatocellular damage is taking place [4, 5].

Drugs, infections, and inflammation in the liver can increase ROS generation and/or decrease GSH levels and cause a shift in the cellular redox status of hepatocytes to become more oxidized. The tripeptide glutathione (GSH) is the most abundant redox molecule in cells and thus is the major antioxidant and redox regulator that is important in combating oxidation of cellular constituents. GSH plays a key role in the detoxification of peroxides, reactive nitrogen species [RNS; e.g., peroxynitrite (ONOO−) and N_2_O_3_], and xenobiotic compounds (such as reactive electrophilic molecules) in cells [6, 7].

The oxidative stress is associated with some natural physiological processes (aging, pregnancy, physical and emotional stress) and also with many diseases and pathological syndromes that have recently been ascribed to a group of free radical related pathologies, including cancer [8]. According to the modern concepts, a pathological condition is associated with the formation of ROS and intensification of free radical oxidation of biomolecules such as proteins, lipids, and nucleic acids [9]. In the cells and tissues, these processes lead to the imbalance between pro- and antioxidant systems accompanied by accumulation of ROS and secondary products of the oxidative modification of biomolecules [10].

The oxidation more readily occurs in lipid structures, primarily in the lipid bilayer of cell membranes and in lipoproteins of blood. As a result of lipid peroxidation (LPO), hydroperoxides, diene conjugates, epoxides, Schiff bases, and aldehydes such as malondialdehyde (MDA) are generating. The excess of LPO products changes the viscosity of the lipid bilayer of the membrane, its composition and surface charge. The intensity of LPO is controlled by endogenous antioxidants, which is extremely important for stabilizing membranes and normalizing cellular metabolism [11].

By acting on macromolecules ROS can alter their structure and function, oxidizing amino acid residues, potentiate DNA damage and alter DNA repair [12]. Oxidized proteins, on the whole, are functionally inactive and more easily subjected to proteolysis. They can accumulate in various tissues, mediating oxidative DNA damage, and can themselves act as a source of free radicals, exhausting the level of cellular antioxidants [13]. In addition to ROS, many other active compounds can be generated (epoxides, aldehydes, ketones, alcohols, dialdehydes, etc.) that may covalently interact with certain functional groups of proteins causing polymerization of proteins and alteration of amino acid residues, especially those containing SH-, SCH_3_-groups of cysteine and methionine, NH-groups of lysine, etc. [14]. Thiols in proteins can undergo a wide range of reversible redox modifications (e.g., S-glutathionylation, S-nitrosylation, and disulfide formation) during exposure to increased concentrations of reactive oxygen and nitrogen species, which can affect protein activity [6]. Thus, the aforementioned processes can lead to protein modifications and conformational changes that also take place with respect to enzymes, affecting their activity.

Among blood plasma proteins serum albumin (SA) is most abundant, the concentration of which yields up to 60% of the total plasma proteins. Despite the fact that ROS, in particular highly harmful hydroxyl radicals (^•^OH), can alter the molecular structure of albumin molecule, SA firmly binds them. Although the damaged albumin molecules are degraded by proteolytic enzymes, their loss is promptly compensated by a newly synthesized portion. In SA the potential target for oxidants is its free SH-group of Cys-34, which is converted into sulfonic group responsible for alterations in protein structure and functions [15].

The method of enterosorption that was proposed in the early 1980s is based on the oral administration of large quantities of specially synthesized adsorbents [16]. Carbonic enterosorbents are widely used in medicine and they were shown to be effective in terms of removal of endo- and exogenous toxins from the body [17]. Moreover, they demonstrated promising results in reducing nausea and vomiting in oncology patients and in achieving positive outcomes in terms of tackling the renal and hepatic failures of various origins, purulent infection, etc. [16, 17, 18, 19, 20]. Enterosorption with carbonic enterosorbents results in significant normalization of a number of biochemical and hematological indexes as well as in enhancement of regenerative processes in detoxifying organs of patients [21].

Recently, in the experiments on mice with implanted Lewis lung carcinoma we demonstrated the definite positive effect of carbonic enterosorption on some paraneoplastic syndrome-associated morphological, hematological and biochemical parameters, and oxidative stress, accompanied by partial plasma protein unloading with recovery of molecular conformation [22, 23].

The main idea of this study is efficacy evaluation of two formulations of activated carbonic enterosorbent in the treatment of rats with severe CCl_4_-induced intoxication. The biophysical approach to study the properties of oxidized plasma proteins provides the qualitative information (melting thermograms) as well as the quantitative information (thermodynamic parameters) about main transport protein such as blood plasma albumin which is important from diagnostic and prognostic points of view.

## 2 Materials and methods

### 2.1 Preparation of carbonic enterosorbents and characteristics

#### 2.1.1 Sample preparation

**Figure 1:**
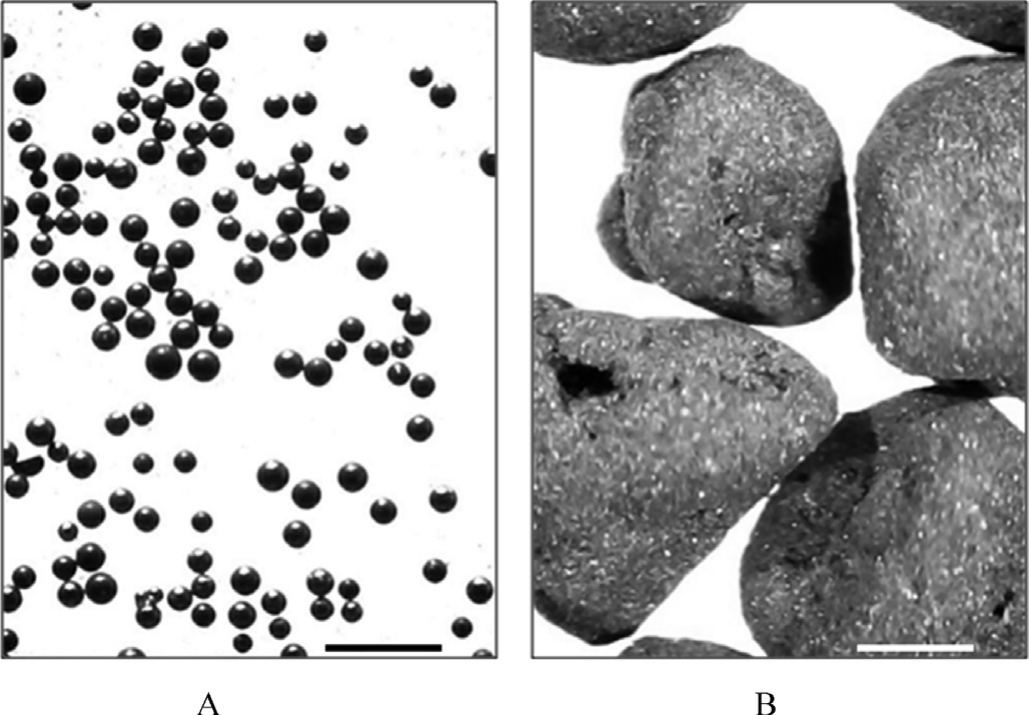
The images of AC1 enterosorbent (**A**) and AC2 enterosorbent (**B**) collected at 20× optical magnification. Scale bars = 1 mm.

Two formulations of activated porous carbonic enterosorbents were derived from carbonized phenol-formaldehyde resin with the surface area of >2200 m^2^/g (as calculated by Brunauer–Emmett–Teller method) obtained from ImmutriX Therapeutics, Inc. (Rapid City, SD, USA). The first formulation of enterosorbent represents the primary solid beads with diameters of 0.15–0.25 mm having the bulk density of 0.16 g/cm^3^ (enterosorbent AC1; Figure 1a). To obtain the second formulation, the carbon beads of AC1 were micronized using an automatic multichannel milling system MKM-300H (“МИЛЛКОМ” Ltd, Kharkiv, Ukraine) followed by gluing the resulting microparticles in starch with subsequent granulation. The final product represented the granules (>1 mm) of irregular shape with the bulk density of 0.13 g/cm^3^ (enterosorbent AC2; Figure 1b).

**Figure 2:**
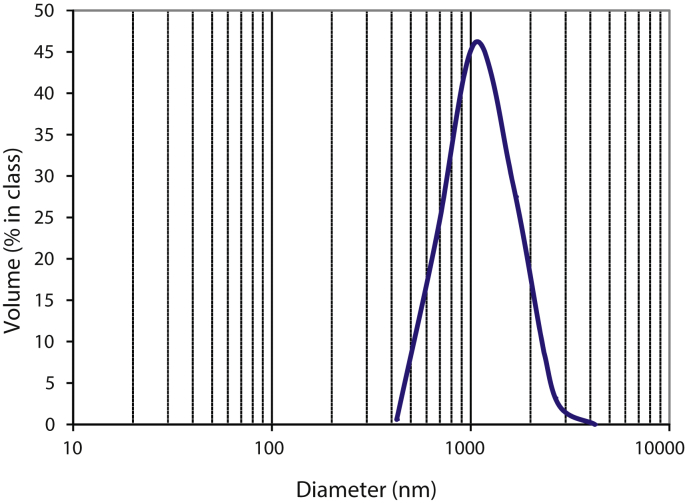
Size distributions of carbon microparticles (from micronized AC1) that were used for production of AC2.

To evaluate the particle size distribution after micronization of AC1, the sample was suspended in pure water (200 μg/mL) and analyzed in 1-mL aliquots by dynamic light scattering using a Malvern Instruments Zetasizer (Worcestershire, UK) equipped with He–Ne laser (25 mW, λ = 633 nm) with an angle detection of 90°. The hydrodynamic diameters of microparticles ranged from 0.42 to 4.21 μm with the mean value of 1.19 μm (Figure 2).

#### 2.1.2 Adsorptive potential of AC1 and AC2 samples

Adsorption of Vitamin B_12_ (B_12_, M.W. = 1355.37 D), creatinine (M.W. = 1355.37 D), unconjugated bilirubin (UB, M.W. = 584.7 D) and methylene blue (MB, M.W. = 373.9 D) by AC1 and AC2 samples was performed in phosphate buffered saline (PBS, pH 7.2) at room temperature and assessed spectrophotometrically using a Synergy HT multi-detection microplate reader (BioTek Instruments, Winooski,VT, USA).

*For B*_*12*_ *adsorption evaluation,* 24 mL of *B*_*12*_ solution (1.0 mg/mL in PBS) was added to 15 mg of the adsorbent and the concentration of *B*_*12*_ was measured at 360 nm.

*For Creatinine adsorption evaluation,* 24 mL of creatinine solution (0.3 mg/mL in PBS) was added to 15 mg of the adsorbent and the concentration of creatinin was measured at 234 nm.

*For UB adsorption evaluation,* 6 mL of *UB* solution (20 mg/dL in PBS solution containing 3 g/dL of human serum albumin, HSA) was added to 15 mg of the adsorbent and the concentration of bilirubin was measured at 456 nm.

For MB adsorption evaluation, 12 mL of MB solution (1.5 mg/mL in PBS) was added to 100 mg of the adsorbent and the concentration of MB was measured at 620 nm.

### 2.2 Design of the experiment

Experiments were performed on adult white inbred female albino rats of 200 ± 20 g weight. The rats were obtained from the IEPOR animal facility (Kyiv, Ukraine). These experiments were performed in the IEPOR according to the contract V-11-17 dated 24 February 2017. The experimental work with animals was approved by the Council of IEPOR Bioethics Commission (protocol № 1a dated 16 February 2017).

Rats (n = 35) were randomly assigned to four groups: Group 1 (n = 10) was treated with CCl_4_; Group 2 (n = 10) and Group 3 (n = 10) were correspondingly treated with AC1 and AC2 enterosorbents two days before and two days after CCl_4_ injections; Group 4 (n = 5) – the intact animals. The 50% solution of CCl_4_ (“Component Reagent” Co, Kyiv, Ukraine) in the extra clean oil was injected subcutaneously at the dose of 0.4 ml per 100 g of rat's body weight once a day for 2 days. The daily amount of both enterosorbents used for administration was 10 cm^3^ per 1 kg of rat's body weight (1600 mg of AC1 and 1300 mg of AC2). These sorbents were fed to the animals in composition with the oatmeal balls twice a day (3 h before and 3 h after the regular meals).

### 2.3 Preparation of samples for oxidative stress assessment

The day after the last enterosorption the animals were sacrificed using general ether anesthesia. The blood samples were immediately processed after heparinization. To obtain hemolysates, the blood (10 μL) was diluted 800 times with distilled water and stored at 3–5 °C before use. Blood plasma was obtained by 3000 rpm centrifugation of blood for 15 min at room temperature followed by storing at -70°С. To study ROS production, 400 μL of blood was centrifuged at 10. 000 g for 30 s and plasma samples were immediately frozen and stored in liquid nitrogen. Liver specimens were removed at 3–5 °C and subsequently stored in liquid nitrogen. Liver homogenates (1:5) were prepared in 0.05 M tris-HCl buffer (pH 7.4) at 3–5 °C. The concentration of proteins in blood plasma and liver homogenates was determined in accordance to the method proposed by Greenberg *et al.* [24].

#### 2.3.1 Assessment of ROS levels in blood plasma

The total level of ROS in blood plasma was largely determined spectrophotometrically in a microplate reader using a N,N-diethyl-p-phenylenediamine (DEPPD) probe as described [25]. DEPPD in acidic media in the presence of oxygen radicals and Fe^2+^ turns into colored form with two peaks in absorption spectrum (λ_abs_ = 511 and 552 nm). Measurements were performed in 96-well microtiter plate using a Sinergy HT microplate reader (BioTek Instruments). Each well contained 5 μL of plasma samples in 145 μL of 0.1 M acetate buffer (pH 4.8) and was incubated at 37°С for 3 min. After this, 100 μl of DEPPD and ferric sulfate in acetate buffer were added (final concentrations – 100 μg/mL and 4.7 μM, respectively) and absorbance at 511 nm was recorded every 0.5 min for 6 min. The content of ROS was calculated from the calibration curve using standard solutions of Н_2_О_2_ (0, 0.37, 0.74, 1.47, 2.21, 2.94, 3.68 and 4.41 mM) and expressed in mM Н_2_О_2_ per 1 L of plasma per min (mM/L/min). The time interval of 1–4 min was considered for calculations, because during this time a colored DEPPD form was generated in a linear manner.

#### 2.3.2 Assessment of reduced glutathione concentration

The reduced glutathione (GSH) content was determined in liver homogenates as described [26] and expressed as nM per mg of protein (nM/mg). For this purpose, the supernatant from the liver homogenates was obtained by centrifugation (6.000 g, 15 min, 5°С). Further, 800 μL of 20% trichloroacetic acid were added to 400 μl of the sample in Tris buffer and incubated at 5–10 °C for 30 min. In the control, distilled water was added instead of the homogenate. Spectrophotometric determination of GSH was performed in 96-well plate, each well was filled with 15 μL of supernatant, 225 μL of 0.3 M Na_2_HP0_4_ (pH 7.7) and 38 μL of 0.4% of 5,5′-dithiobis(2-nitrobenzoic acid; DTNBA). Optical densities of the samples were measured at 412 nm following 20 min incubation at room temperature after the addition of DTNBA.

#### 2.3.3 Measurement of catalase activity

The activity of antioxidant enzyme catalase (CAT) was determined in the blood hemolysate of experimental rats by the method based on the properties of H_2_O_2_ to form a stable colored complex with the salts of molybdenum [27]. This method was adapted for the analysis in a plate reader. The reaction was conducted in test tubes containing 0.8 mL of 20 mM H_2_O_2_ and 40 mkl of sample. After 10 min the reaction was stopped by adding 0.4 mL of 4% ammonium molybdate ((NH_4_)_2_MoO_4_) followed by transferring of 0.25-mL aliquots in 96-well plate and measuring the optical density at 410 nm using a Sinergy HT microplate reader (BioTek Instruments). The amount of utilized Н_2_О_2_ was assessed with a calibration curve (0, 5, 10 and 20 mM Н_2_О_2_), and the CAT activity was calculated as mM Н_2_О_2_ per mL of blood per minute (mM/mL/min).

#### 2.3.4 Assessment of pro/antioxidant ratio

The intensity of free radical processes (FRP) was evaluated by determining the pro/antioxidant ratio in blood by the method of Н_2_О_2_-induced chemiluminescence [28]. The total amount of light emitted in 3 min of the reaction, reflecting the pro/antioxidant ratio of the chemical products in the sample.

#### 2.3.5 Assessment of the oxidative damage of lipids

The level of lipid peroxidation (LPO) was studied by evaluating the content of MDA in the liver homogenate and blood plasma by the method based on the ability of MDA to form a stable and colored trimethine complex with 2-thiobarbituric acid [29, 30].

#### 2.3.6 Determination of oxidative modification of proteins

The level of the oxidative modification of proteins (OMP) was estimated by Levine *et al.* method [31] modified by Dubinina *et al.* [32]. The method based on the reaction of proteins oxidized amino acid residues with 2,4-dinitrophenylhydrazine (2,4-DNPH) with subsequent formation of its derivatives. By spectrophotometric analysis the following 2,4-DNPH derivatives were investigated: aldehyde-dinitrophenylhydrazone (A-DNPH, at 274 nm), and ketone-dinitrophenylhydrazone (K-DNPH, at 370 nm). To obtain a coherent view on oxidation of proteins in blood plasma, spontaneous and metal-catalyzed OMP were studied as well.

### 2.4 Estimation of conformation changes of plasma proteins by biophysical methods

Melting thermograms were recorded on a DASM-4 microcalorimeter (Biopribor, Russia) at a scanning rate 1 °C/min. Steady state fluorescence spectra were recorded on a ND3330 fluorospectrometer (NanoDrop, USA). The experiments were performed at 25 °C.

### 2.5 Statistical analysis

The data were expressed as the mean ± standard error of the mean. The probability valueswith p < 0.05 were considered as statistically significant. The distribution of the analyzed data was estimated using the Shapiro–Wilk normality test. To assess the statistical significance of the differences between means, the Mann–Whitney U test and ANOVA test were applied using Origin Pro 7.5 software (OriginLab Corporation, Northampton, MA, USA).

## 3 Results

**Figure 3:**
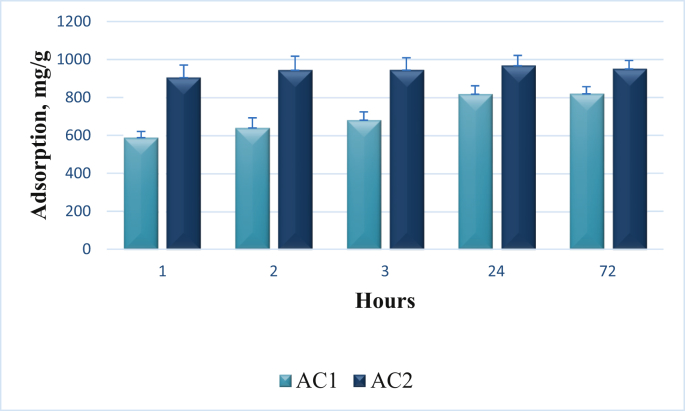
Kinetic data of MB adsorption by primary (AC1) and secondary granules (AC2) of carbonic enterosorbents, p < 0.05.

The kinetic data of methyl blue (MB) adsorption from its water solution are presented in Figure 3. These data clearly show that despite the excellent transport porosity of the primary beads of activated carbon (AC1), the equilibrium point of the dye concentration for these beads was reached on the 2nd day after adsorption was started. In contrast to AC1, the equilibrium point for the secondary granules of activated carbon (AC2) was reached much faster (i. e., within 2 h after adsorption was started). This obvious difference in the adsorption kinetics qualities of primary and secondary carbon beads or granules may hypothetically be a basis for their different biological and curative properties.

**Figure 4:**
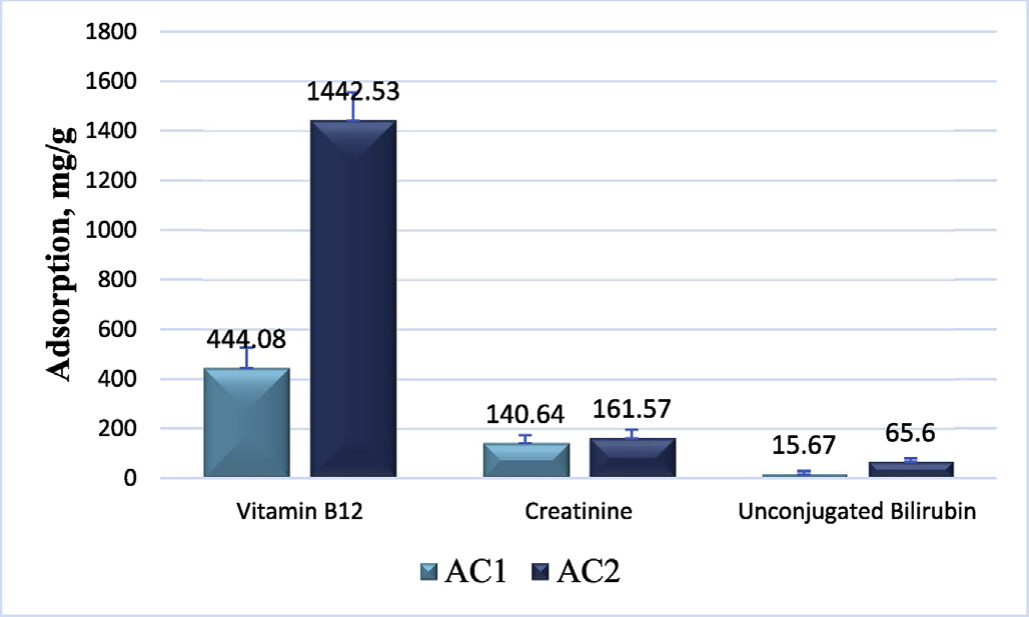
Vitamin B_12_, creatinine and unconjugated bilirubin adsorption by primary (AC1) and secondary granules (AC2) of carbonic enterosorbents, p < 0.05.

To test this hypothesis, we studied the adsorptive potential of enterosorbents towards vitamin B_12_ (represents a middle molecular weight substance), creatinine (represents a water-soluble metabolite), and unconjugated bilirubin (represents a tightly protein-bound substance of hydrophobic nature), as shown in Figure 4.

Figure 4 demonstrates that indeed, the adsorptive potential of the secondary granules of the enterosorbent AC2 significantly exceeds that for AC1 in all, without exception, tested biologically active marker substances.

In rats exposed to CCl_4_, the administration of AC2 (Group 3) resulted in a significant (p < 0.05) intensification of the ROS formation in blood plasma by 17.6% compared to Group 1. Compared to intact rats (Group 4), the increase in ROS formation was 19.9%, but the difference between the groups had a character of a tendency only due to the greater variability of the individual parameters for group 4. The use of AC1, compared to AC2, had an opposite effect. The level of ROS formation was by 13.0% lower than that in control (Group 4) and significantly lower than that in group 3 (p < 0.05, 1.38-fold).

**Figure 5:**
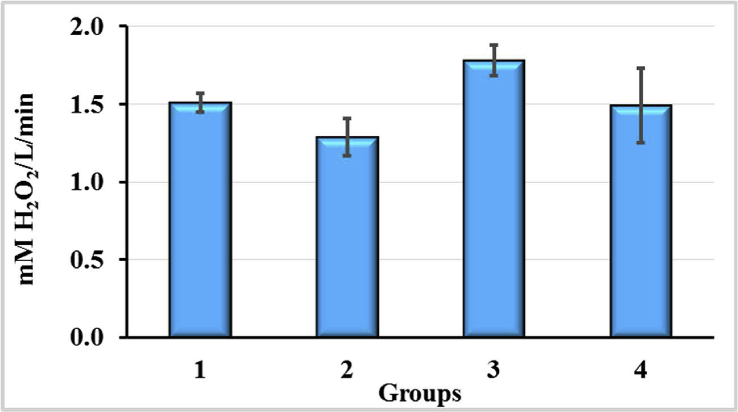
The intensity of reactive oxygen species (ROS) formation in blood of rats exposured to CCl_4_ with or without enterosorption using AC1 and AC2 formulations, p < 0.05.

The results of the intensity of ROS formation study in blood plasma of experimental animals are shown on Figure 5. After injections, CCl_4_ did not lead to significant changes in ROS production. However, the ROS production effects of CCl_4_ in combination with AC1 and AC2 were significant. AC1 reduced the formation of ROS, whereas the effect of AC2 was opposite. It should be noted that the maximum changes in comparison with intact animals did not exceed 20%.

**Figure 6:**
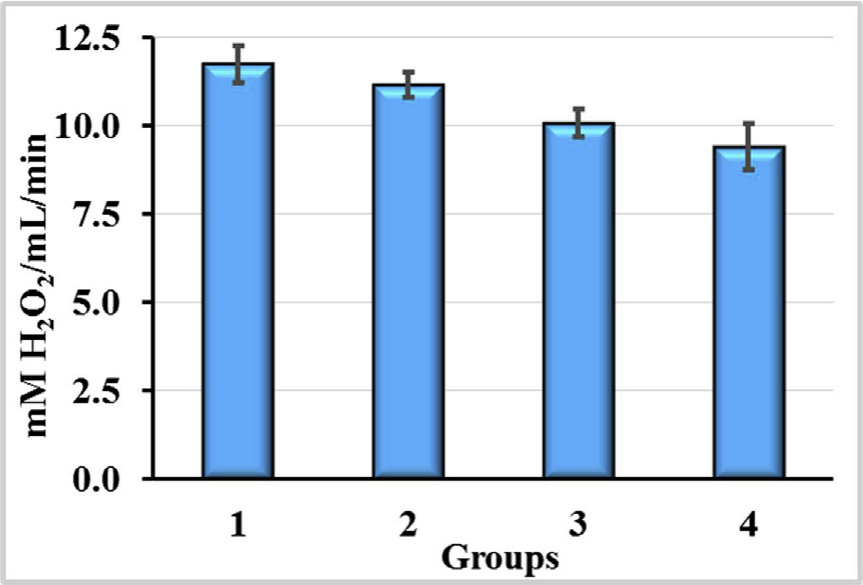
Catalase (CAT) activities in blood of rats exposured to CCl_4_ with or without enterosorption using AC1 and AC2 formulations, p < 0.05.

The results of the CAT activity study in blood of experimental animals are shown on Figure 6. After injections, CCl_4_ treatment resulted in a significant increase of CAT activity in blood by 24.7%. The use of enterosorbents on the background of the action of CCl_4_ led to the normalization of CAT activity. In the case of AC2, the effect was more pronounced. In rats of this group, the activity of the enzyme in the blood did not significantly differ from the control but was obviously lower than in group of animals treated with CCl_4_ (1.16-fold, p < 0.05). In the case of AC1, the activity of CAT remained elevated and exceeded its activity in the control by 18.5% (p < 0.05).

CAT activity under the influence of AC2 is more close to normal than under the influence of AC1. Thus, CCl_4_ in the animals caused the changes in CAT activity. As a consequence of the action of the enterosorbents AC1 and AC2, the CCl_4_-induced activation of CAT has been decreased. However, the effects of AC1 and AC2 were not identical. In the case of AC1, the activity of the enzyme remained significantly increased, and under the conditions of AC2 use, it did not differ much from the activity of CAT in blood of intact rats.

**Figure 7:**
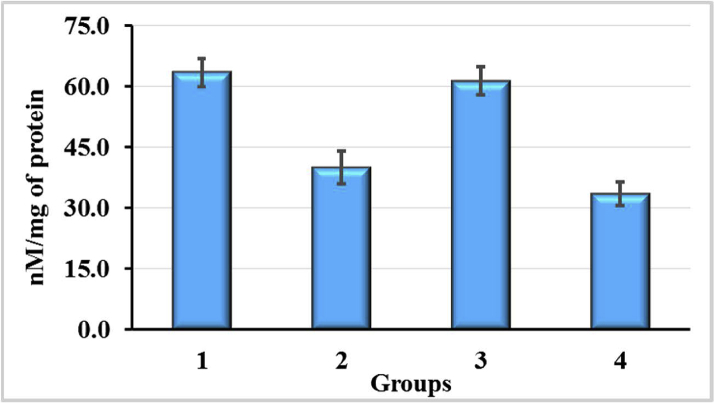
Reduced glutathione (GSH) content in liver of rats exposured to CCl_4_ with or without enterosorption using AC1 and AC2 formulations, p < 0.05.

The results of the GSH content analysis in the liver of rats after exposure to CCl_4_ and the use of enterosorbents AC1 and AC2 are presented on Figure 7. Treatment animals with CCl_4_ resulted in significantly increased levels of GSH in the liver (1.89-fold, p < 0.05). The effect of reducing the concentration of GSH due to the use of the enterosorbents was more pronounced in the case of AC1. Here, the level of GSH 1.19-fold exceeded that in control group 4 (p > 0.05), but was significantly lower than in CCl_4_ group 1 (p < 0.05). Contrary to AC1, AC2 did not significantly affect the level of GSH if compared with CCl_4_ group 1, although its content was significantly higher (p < 0.05) compared to the control group 4 (1.83-fold) and group 2 exposed to CCl_4_ and AC1 (1.53-fold). Thus, there were drastic differences in liver GSH contents of CCl_4_-exposured animals depending on the type of activated carbon used for tackling CCl_4_-associated intoxication. While AC1 led to a sharp decrease in the content of GSH in the liver and its level was slightly different from that of control, AC2 had almost no influence on this index and it remained significantly elevated. It should be noted that such a remarkable difference in the effects of AC1 and AC2 in combination with CCl_4_ was also observed with respect to the formation of ROS in blood plasma (see Figure 5).

The total level of intensity of free radical oxidation processes in body tissues and an efficiency of its inhibition by enzymatic and non-enzymatic antioxidant system displays pro/antioxidant ratio, which is quite labile integral indicator and shows the balance of prooxidant and antioxidant processes. Disturbance of this ratio due to the formation of free radicals and organic compounds is one of the major mechanisms of formation of structural and functional damage and the development of oxidative stress, which is accompanied with the accumulation of ROS and secondary products of oxidative modification of biomolecules that belong to different classes of organic compounds (proteins, lipids, nucleic acids), in tissues and biological fluids.

**Figure 8:**
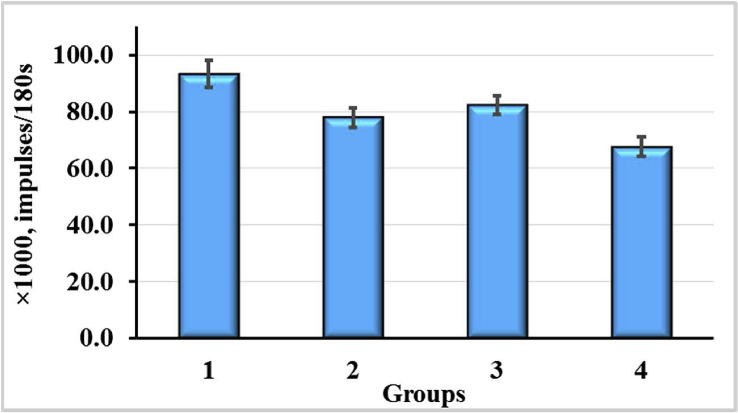
Pro/antioxidant ratio in peripheral blood of rats exposured to CCl_4_ with or without enterosorption using AC1 and AC2 formulations, p < 0.05.

According to the results of our study, there was a significant increase (by 27.5%, p < 0.05) of the pro/antioxidant ratio in the blood of rats treated with CCl_4_ as compared with the control values (Figure 8). If the enterosorbents were used, a decrease in this ratio in the blood of rats was observed in the direction of approaching to the control values. Enterosorbent AC1 reduced the intensity of free radical oxidation processes in blood of rats by 16.5% (p < 0.05) and AC2 - by 11.7% compared to the CCl_4_ group.

Level of correction of pro/antioxidant ratio in peripheral blood under the influence of AC1 and AC2 was the same. Thus, CCl_4_ caused an increase in the prooxidant processes in the animal organism, leading to the oxidative stress development. The detoxification efficiency of carbon enterosorbents depended on the impact of the damaging factor on the animals and is practically the same for AC1 and AC2.

It is supposed that in the state of oxidative stress, the attack of free radicals is primarily targeted not on the lipids, but on the proteins of the plasma membranes. The confirmation of the primary peroxidation of the proteins under oxidative stress is the presence of pronounced changes of anulic lipids, which suggests the leading role of OMP in the destruction of the cell membrane. The most common type of protein damage is the formation of carbonyl groups of proteins when they are oxidized and aldehyde and ketone groups of amino acid residues are formed. The processes of peroxidation of proteins are indicated by the accumulation of protein degradation products in the form of A-DNPH and K-DNPH, which are markers of OMP, in the investigated material. Two indices are used in the determination of OMP: spontaneous and induced (metal-catalyzed). The first index characterizes the constitutive activity of the OMP, the second indicates the amount of substrate for oxidation and the possibility of its involvement in these processes. It can be considered as an indicator of system stability before peroxidation.

**Figure 9:**
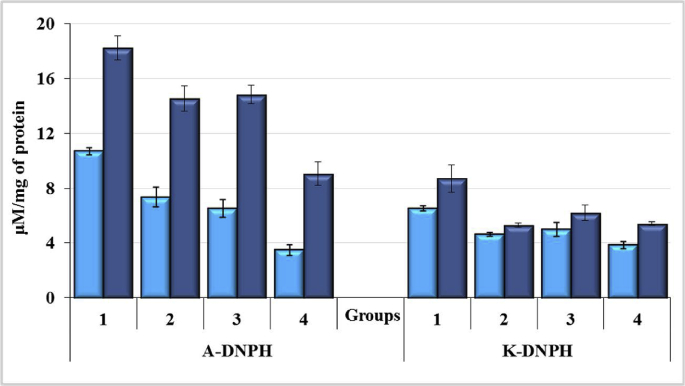
The level of oxidative modification (spontaneous  and induced  levels) of blood plasma proteins in rats exposed to CCl_4_ with or without enterosorption using AC1 and AC2 formulations, p < 0.05.

In the model of acute liver failure in rats after administration of CCl_4_, the modifying effect of enterosorbents AC1 and AC2 on the level of OMP in the blood and liver of experimental animals was investigated. The action of CCl_4_ led to a significant increase in both spontaneous and induced OMP plasma levels, which significantly differed from the control values (Figure 9). Thus, the spontaneous levels of A-DNPH by 3.1 times, and K-DNPH by 1.7 times exceeded (p < 0.05) their values in plasma of blood of intact animals. The use of the carbonic enterosorbents significantly lowered the toxic effect of CCl_4_: the spontaneous level of A-DNPH in rat plasma decreased by 1.5 and 1.6 times, and K-DNPH by 1.4 and 1.3 times under the action of AC1 and AC2, respectively. The induced A-DNPH level in blood plasma was more significant, which in intact animals exceeded the spontaneous level of OMP by 2.6 times, and by 1.7 times in the case of action of CCl_4_. AC1 and AC2 significantly lowered the induced level of A-DNPH (1.3- and 1.4-fold, respectively) and K-DNPH (1.6- and 1.4-fold, respectively). Induced levels of A-DNPH and K-DNPH under the action of CCl_4_ by 1.8 and 1.3 times exceeded their values in the control group. Under enterosorbents action, the level of oxidative damage of liver proteins was lowered (A-DNPH by 1.6–1.5 times, K-DNPH by 1.3–1.2 times) up to the control level (Figure 9).

**Figure 10:**
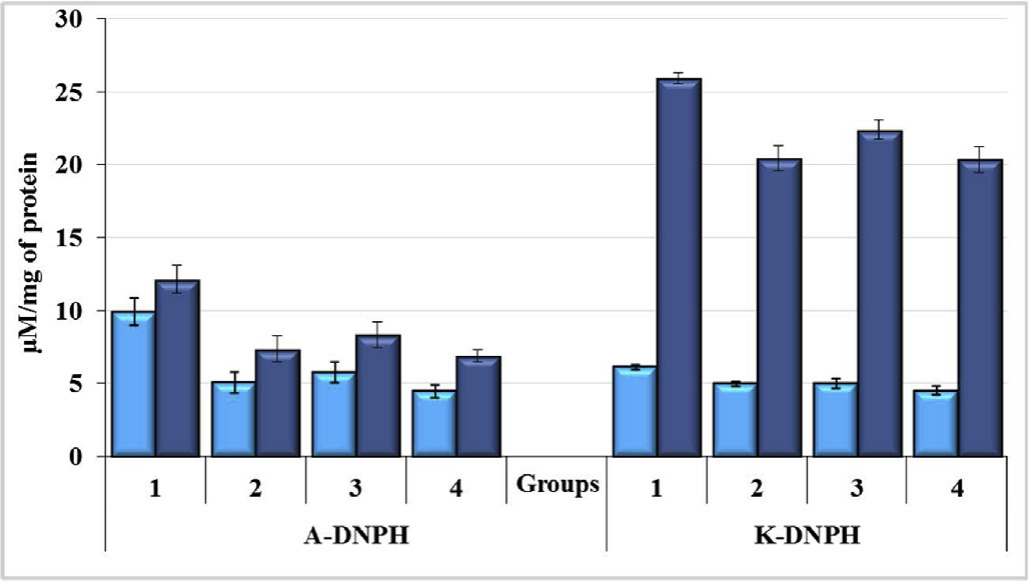
The level of oxidative modification (spontaneous  and induced  levels) of proteins in liver homogenates of rats exposed to CCl_4_ with or without enterosorption using AC1 and AC2 formulations, p < 0.05.

Thus, significant differences in the levels and forms of OMP in blood plasma of rats were observed. The toxic effect of CCl_4_ increased both spontaneous and induced levels of OMP, and the action of the enterosorbents reduced the degree of oxidative damage of plasma proteins without affecting the system's stability for peroxidation. Between four parameters of oxidative modifications of blood proteins two - are closer to normal under the influence of AC, one – under the influence of AC2, another ones gave equal changes for AC1 and AC2. Taking into account the hepatotoxic nature of CCl_4_, its effect was accompanied by an increase in the generation of products of oxidative modification of liver proteins. The spontaneous levels of A-DNPH and K-DNPH in rats exposed to CCl_4_ increased 2.2- and 1.4-fold, respectively, compared to intact animals. AC1 and AC2 significantly lowered the content of A-DNPH (2.0- and 1.7-fold respectively), though for K-DNPH, both formulations caused moderate reduction of its content (1.2-fold, p > 0.05), compared to the control (Figure 10).

Among four parameters (Spontaneous and Induced Levels) of OMP in rat liver homogenates, three were closer to normal under the influence of AC1 and the fourth parameter (KPDH, Spontaneous Level) had the same improvement for AC1 and AC2. Thus, the hepatotoxic agent CCl_4_ greatly enhanced the spontaneous and induced levels of OMP in the blood plasma and liver of rats, which is an early manifestation of oxidative damage to macromolecules in the body. A comparative study of the effects of AC1 and AC2 enterosorbents on OMP levels in animals with acute hepatic insufficiency revealed their ability to indirectly reduce the level of oxidative damage to proteins in the blood and liver of experimental animals, thus preventing the development of oxidative stress in the body. Most effective in the normalization of OMP level was found AC1.

Deserves attention the significant (more than 4-fold) increase in the induced level of K-DNPH in liver proteins both in the control group and animals administered with hepatotoxic agent CCl_4_, whose metabolism is predominantly occurring in the liver. This may indicate significant reserves of the liver protein system to the peroxidation, as well as the accumulation of oxidative damage of proteins under the action of CCl_4_, which is an early manifestation of oxidative stress development.

**Figure 11:**
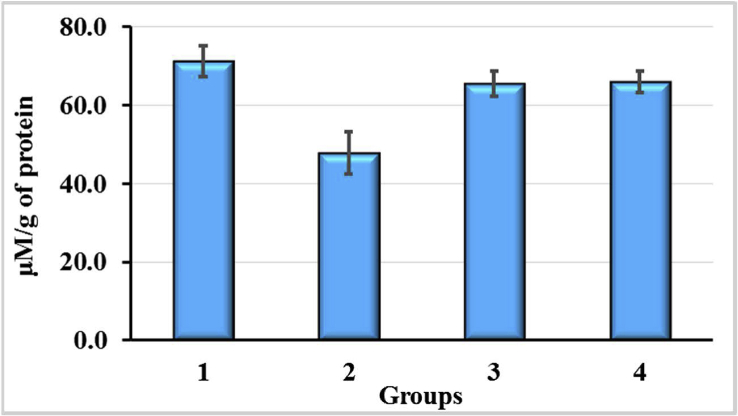
Malondialdehyde (MDA) levels in the blood plasma of rats exposured to CCl_4_ and enterosorbents, p < 0.05.

**Figure 12:**
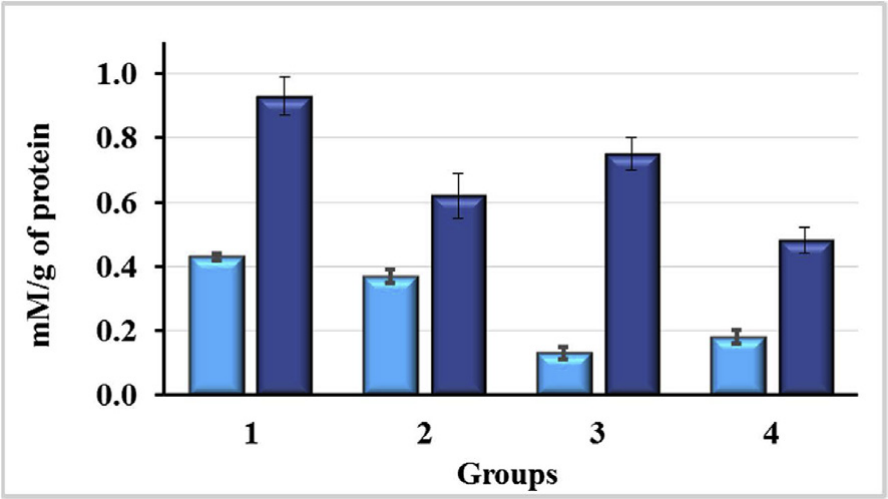
Spontaneous (mM/g protein) and induced (mM/g protein/30 min) levels of MDA in rat liver homogenates after exposure of animals to CCl_4_ and enterosorbents, p < 0.05.

Peroxide oxidation products have high reactivity and are quite toxic, which enhances the course of the pathological process. The secondary product of peroxidation is MDA, its content reflects the level of the LPO. The results of the studies on the content of MDA in blood and liver of rats are presented in Figures 11 and 12, respectively. While studying the process of LPO in blood plasma of rats, a similar tendency of changes was observed as in the study of the pro/antioxidant ratio in blood. There was a small increase in the level of MDA due to the negative effect of CCl_4_ (Figure 11).

The effect of AC1 on animals exposed to CCl_4_ resulted in a 33% reduction in MDA content (p < 0.05), which is even lower than in control animals. Such a decrease in the content of MDA caused by enterosorbents relates to the induction of protective antioxidant systems, or the direct effect of the sorbent itself, targeted on inactivation (decomposition) of MDA. The MDA content in rat plasma decreased more readily under the influence of AC1 enterosorbent then after administration of AC2 when its level was similar to that of intact rats.

The study of the LPO in the liver of rats (Figure 12) showed that the effect of CCl_4_ caused a significant increase in the background level of MDA (by 138%; p < 0.05). The use of enterosorbents lowered MDA level in liver. AC2 was found to be more effective reducing the background level of MDA by 70% (p < 0.05), whereas AC1 reduced it just by 14% (p < 0.05).

Spontaneous and induced levels of MDA in rats liver homogenate were closer to the normal under the influence of AC2 enterosorbent in comparison with AC1. The nature of the changes of the level of induced TBA-active products, supporting the presence of such products in the system before oxidation, is the same as the background ones. Significant increase in the induced level of MDA in the liver homogenate under the action of CCl_4_ by 93.7% (p < 0.05) may indicate a profound disorder of oxidative metabolism in the rats liver. AC1 reduced the content of MDA by 33.3% (p < 0.05), and AC2 by 19.4% (p < 0.1). Thus, CCl_4_ caused the intensification of the processes of LPO in blood plasma and liver. Enterosorbents led to a decrease in the level of MDA, in some cases, even below its level in intact animals.

**Figure 13:**
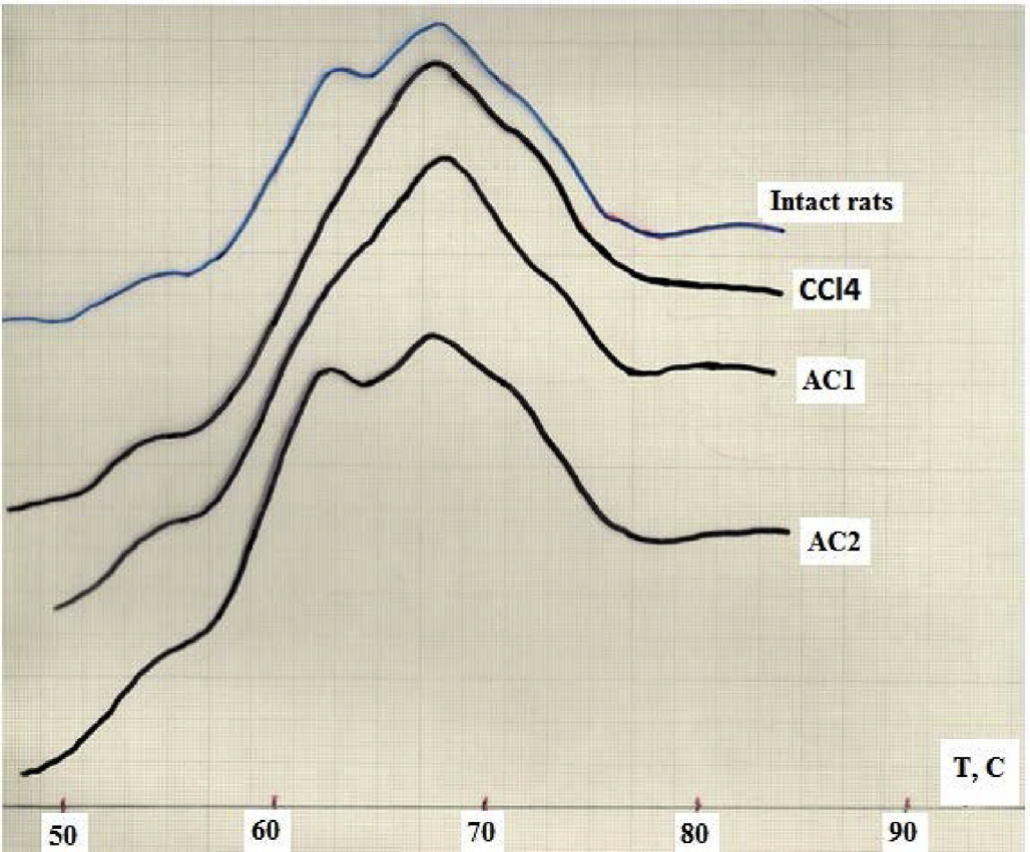
Melting thermograms of blood plasma proteins of rats exposed to CCl_4_ with or without enterosorption using AC1 and AC2 formulations, compared to that of intact animals.

The melting temperature of blood plasma proteins (BPP), serving as an integral criterion for degree of their ligand loading, has been previously reported [33]. In this study, the comparison of melting thermograms of BPP showed the differences in their shape and positions of peaks (Figure 13). The melting curve for BPP of intact rats (group 4) representing by three clearly defined peaks in the region of 53 ± 1, 62 ± 1 and 69 ± 1 °C, the second maximum belongs to albumin fraction, which is dominant among plasma proteins. In CCl_4_-exposed rats, there were visible changes in the melting curves of serum albumin. Maximum observed at 62 ± 1 °C shifts to a higher temperature region at 70 ± 1 °C (group 1). This indicates an increase of ligand loading of this protein by endogenous metabolites and toxins [34], the level of which increases in response to injection of this hepatotoxin. In the case of AC1 administration (group 2) there was a tendency toward the appearance of the thermodenaturation maximum within 62–63 °C, while in the case of AC2 administration (group 3) there were a distinct maximum at 62.4 °C and a qualitative change in the shape of melting thermogram similar to that of intact animals (group 4). As a result of such type of serum “cleansing” under the influence of AC2 administered into gastro-intestinal tract, albumin molecules can gradually recover their native conformation [35].

**Figure 14:**
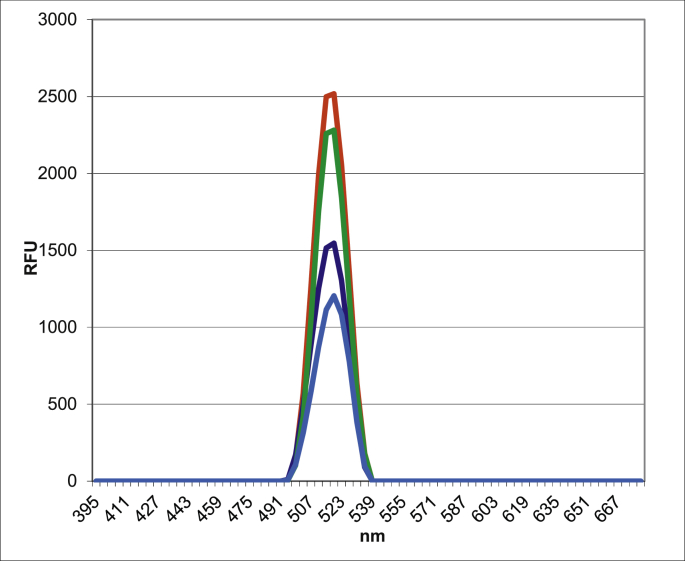
Intrinsic fluorescence characteristics of blood plasma albumin of intact rats (red), CCl_4_-exposed rats (blue), CCl_4_-exposed rats that received AC1 (violet) and AC2 (green).

In addition to the recovery of calorimetric profiles of BPP, the intrinsic fluorescence characteristics of serum albumin can also tend to be recovered, gradually approaching those of intact animals (Figure 14). Return to the native molecular conformation of albumin can be explained by the removal of protein-bound toxins from its surface, revealing the relative changes in the proximity between amino acid residues attributing to the active fluorescence emission of the protein. Otherwise, any metabolite or toxin bound to albumin can result in quenching, a scenario that may take place due to CCl_4_ intoxication.

The fluorescence spectra of blood plasma albumin of rats from all four groups presented in Figure 14. The figure shows a significant decrease of blood plasma albumin fluorescence in CCl_4_-exposed rats compared to that in intact rats. Treatment of CCl_4_-exposed animals with enterosorbents, particularly with AC2, resulted in significant increase of fluorescence emission of albumin molecules, suggesting recovery in their conformation.

## 4 Discussion

The motivation to use the method of enterosorption was based on multiple positive results of *per os* usage of carbonic adsorbents for therapy of a number of pathologies including acute radiation sickness, chronic glomerulonephritis, biliary and portal cirrhosis, severe forms of leptospirosis, food allergy, psoriasis, acute enteric infections, idiopathic dilation cardiomyopathy, and chronic renal failure, as well as severe myelo-, nephro-, cardio- and gonadotoxic side effects of intense cancer chemotherapy [36, 37, 38, 39, 40, 41, 42, 43]. All these reports evidenced on significant positive therapeutic pathomorphosis of mentioned pathologies under the influence of enterosorption, first of all, on acceleration of reparative processes in damaged organs and tissues. The main mechanisms of action of enterosorption, which explain their restorative effects, are the adsorption of different exogenous and endogenous toxins, among them the products of lipid and protein peroxidation. Adsorbents can bind toxins from systemic bloodstream and internal media by direct diffusion from the blood and/or digestive juices. Additionally, enterosorbents can indirectly stimulate the exchange and excretion of toxins by detoxifying organs, as well as binding and transporting biologically active substances to the surface of enterosorbent [44].

Different adsorptive potential of the two formulations of enterosorbents were represented in Figures 3 and 4 which demonstrate a significant advantage of micronized enterosorbents AC2 for all marker substances, including methylene blue, vitamin B_12_, creatinine and unconjugated bilirubin, supporting the hypothesis of their different biological and therapeutic qualities. Despite the difference in adsorptive parameters both enterosorbents possesses very high protective potential. It should be noted that powder enterosorbents were found to be absolutely non aggressive for gastrointestinal tract (GIT) mucosa. Earlier we have demonstrated that after two-week long enterosorption session, the state of GIT of experimental animals was similar to that of intact mice and in histological structure of fundic gastric glands, villi and crypts of small intestine (duodenum), and large intestine mucosa (rectum) no pathologic alterations or abnormal inclusions were observed that evidenced on high safety and proper evacuation of the enterosorbents from an organism of experimental animals [45].

This study designed to evaluate a protective effect of two formulations of carbonic enterosorbents: primary solid beads (AC1) and micronized form (secondary granules, AC2) under conditions of rat liver injury achieved by the administration of CCl_4_. This liver toxin caused oxidative and nitrosative stress accompanied by an increase in ROS and RNS generation, GSH level change and cause a shift in the cellular redox status of hepatocytes to become more oxidized. These changes in turn affect conformation of blood plasma proteins, the values of pro/antioxidant ratio, MDA levels and oxidative protein modification. GSH guards cells against oxidative injury by reducing H_2_O_2_ and scavenging reactive oxygen and nitrogen radicals that can oxidize macromolecules and damage cells. The observed high level of GSH in Group 1 animals was a compensation reaction of the organism on CCl_4_ effect. The effects of AC1 and AC2 use at the background of the CCl_4_ action were significantly different. If AC1 sharply reduced the content of GSH, and its level in the liver exceeded the control just by 1.19 times, AC2 exerted weak influence on this index, and it remained elevated by 1.83 times. GSH is the most abundant redox molecule in cells and thus the most important determinant of cellular redox status. An alteration of the normal redox balance can alter cell signaling pathways in hepatocytes and may thus be an important mechanism in mediating the pathogenesis of many liver diseases.

GSH-induced redox shift with or without ROS subjects some cellular proteins to varied forms of oxidation, altering the function of signal transduction and transcription factor molecules [7]. Protein oxidation may entail a wide range of chemical modifications including hydroxylation of amino acids, such as tyrosine and phenylalanine hydroxylation, methionine oxidation to methionine sulfoxide, and oxidation of thiols in cysteine residues to disulfides. The thiols in cysteine residues of proteins can undergo various reversible and irreversible redox alterations in response to ROS and RNS stress that may be a central mechanism of protein regulation [6]. The comparative study of the effect of AC1 and AC2 enterosorbents administration on oxidative modification of plasma and liver proteins confirmed their capability to reduce indirectly the intensity of OPM, and consequently prevent the development of side effects of oxidative stress induced by CCl_4_. Between four parameters of oxidative modification of proteins in rat liver homogenates three – are closer to normal under the influence of AC1 and last one gives the same level of amelioration for AC1 and AC2. Indeed, in this study, MDA content in rat plasma also decreased more effectively under the influence of AC1 enterosorbent. Still, result of AC2 usage completely coincided with intact rats MDA level. Spontaneous and induced levels of MDA in rats liver homogenates were closer to the normal under the influence of AC2 enterosorbent in comparison with AC1. However, extent of correction of pro-oxidant/antioxidant ratio in peripheral blood under the influence of AC1 and AC2 was the same.

**Figure 15:**
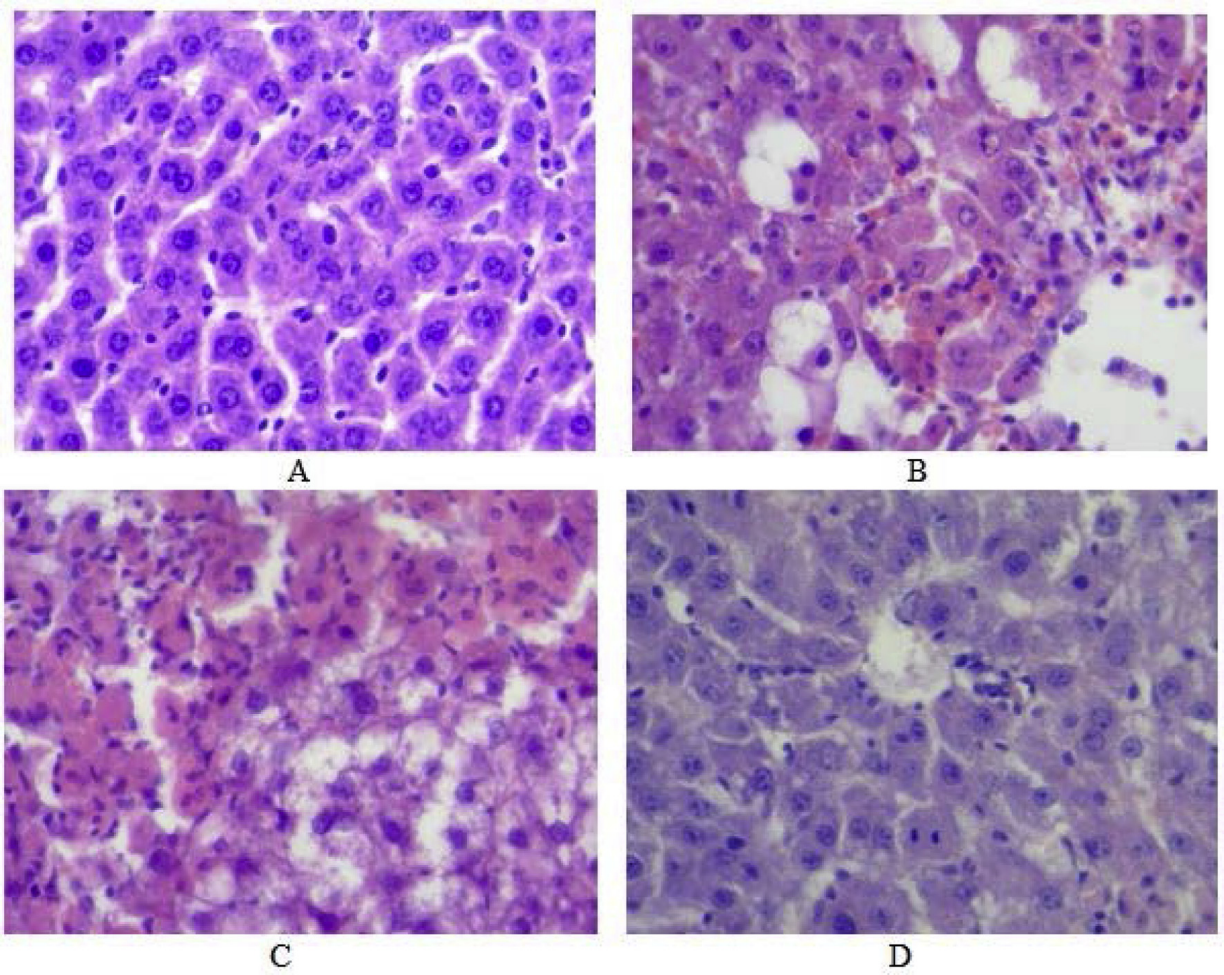
Histology of the liver of (A) intact control rat; (B) CCl_4_-group. Full-blooded sinusoids with erythrostasis at centrilobular zone. Large-droplets acute toxic fatty degeneration (dystrophy) of centrilobular hepatocytes; (C) rat from CCl_4_ + AC1 group. Hyaline-droplet protein dystrophy of hepatocytes and (D) CCl_4_ + AC2 group. Local narrowing of central vein's lumen, hyaline-droplet protein dystrophy. Stained with hematoxylin and eosin. Magnification: 200×.

The detected opposite effects of the enterosorbents in different body systems, probably, related to a different mechanism of their action. In particular, AC1 under the action of CCl_4_ is the more effective detoxificant in blood plasma, while AC2 - in liver. This phenomenon was confirmed by the data of histological examination of the liver of rat, exposed to CCl_4_, demonstrated acute toxic damage. Central veins were dilated and less-blooded (Figure 15A), sinusoids were not visualized. The classical lobular structure of liver was disrupted: the bulk organization of hepatocytes was lost; intercellular contacts were damaged. Centrilobular hepatocytes experienced large-droplets acute toxic fatty dystrophy (Figure 15B) with local lymphoid infiltrates.

The cytoplasm of most cells has signs of hyaline-droplets protein dystrophy, local karyopicnosis, and karyolysis. Sinusoids' lumens of central lobule area contain erythrocytes, what is the sign of stagnant phenomena. The reaction of vessels of portal tracts was minimal. There are the sings of intra-duct cholestasis. There was no significant change in liver histology in case of AC1 correction of CCl_4_-damage, except narrowing of central veins' lumens (Figure 15C). However, AC2 demonstrated pronounced tendency to improve the morphological structure of the liver in CCl_4_ acute toxicity. Under it influence bulk organization of the liver was partially and locally restored, but the lobular structure and intracellular contacts were still hardly damaged (Figure 15D), sinusoids’ lumen did not visualize almost. Large-droplet fatty dystrophy was observed in centrilobular hepatocytes, but its manifestations were less severe.

It is highly likely that above-mentioned results are associated with positive changes of serum albumin molecular conformation in rats treated with AC2 enterosorbent. Earlier we demonstrated that enterosorption therapy allows to decrease the signs of systemic intoxication and improves the detoxification potential of organism in rats with Levis lung carcinoma [46] including kidneys and liver functions. Recovery of liver functions assumes an increase the synthesis of serum albumin with a normal structural and functional activity and the utilization of oxidized forms of this protein. It should be noted that apart from the protein concentration, the most important index is the content of “effective” albumin fraction, i.e. the fraction with restored binding and transporting properties. However, the oxidative modification of proteins which occurs upon the influence of reactive oxygen species often prevents normal functioning of this protein. An intensity of this process is predetermined by amino acid composition of proteins and could be considered as an early marker of oxidative stress. The degree of oxidative modification of albumin can be estimated by the concentration of mercaptalbumin and non-mercaptalbumin or their ratio, or by determination of albumin ability to bind cobalt iones (Albumin Cobalt Binding Test) [46, 47]. However, both of these methods do not give the information about the conformational changes in this protein in response to the oxidation process. For this reason, two biophysical methods such as differential scanning microcalorimetry and fluorimetric analysis were used for integral assessment of structural changes in albumin molecule caused not only by oxidation process but also overloading with hydrophobic and amiphilic metabolites and toxins accumulated in blood plasma due to liver insufficiency caused by injection of hepatotropic poison.

These biophysical methods also turned out to be very informative for evaluating the effectiveness of both enterosorbents formulations. Thus, the administration of AC2 enterosorbent, with a very high adsorptive potential, demonstrated more pronounce “internal” purification of rat serum albumin than AC1, and partial restoration of the native molecular conformation of this protein.

The observed differences in experimental outcomes data cannot be fully explained by the different adsorption capacity of the AC1 and AC2 adsorbents. The primary beads and secondary granules of carbonic enterosorbent also have different pharmacokinetic and organoleptic properties. The fine carbon fraction possesses a significantly larger external surface area compared to granules and, correspondingly, a higher sorption-kinetic potential, which was evaluated by adsorption of marker compounds under standard conditions. Moreover highly dispersed aqueous suspensions of powdered enterosorbents greatly facilitate the process of their oral administration to small animals and make it practically non-traumatic. Solid beads retain their adsorptive activity at the lower part of the intestine, while orally disintegrating micronized forms undergo fast saturation starting in the oral cavity. No pathologic alterations or abnormal inclusions were observed that evidenced on high safety and proper evacuation of the enterosorbents from an organism of experimental animals.

## 5 Conclusions

Oxidative stress is a key factor contributing to pathogenesis of acute liver failure. The use of carbonic enterosorbents AC1 and AC2 at the background of the action of CCl_4_ led to their opposite effects – AC1 lowered the intensity of ROS formation and AC2 increased it, however, the difference in intensity of ROS from intact animals did not exceed 20%. Administration of CCl_4_ in rats led to an increase of the CAT activity in the blood and a 1.89-fold increase in the GSH content in the liver of experimental animals compared to the intact rats. AC1 sharply reduced the content of GSH, and its level in the liver exceeded the control just by 1.19 times, AC2 exerted weak influence on this index, and it remained elevated by 1.83 times.

The effect of CCl_4_ resulted in an increase in the anti/prooxidant ratio in rat hemolysates by 27.5%. Level of correction of prooxidation ratio in peripheral blood under the influence of AC1 and AC2 were the same. MDA content in rat plasma decreased more effectively under the influence of AC2 enterosorbent. Still, result of AC2 usage completely coincided with intact rats MDA level. Spontaneous and induced levels of MDA in rats liver homogenates were closer to the normal under the influence of AC2 enterosorbent.

Positive influence of micronized porous carbon enterosorbent AC2, used as the secondary grains, on some biophysical indexes of blood plasma proteins of experimental animals poisoned with carbon tetrachloride were established. This adsorbent helps to restore natural detoxification potential of organism through deliganding serum proteins (in particular – albumin) from endogenous metabolites and toxins and the recovery of the native conformation of albumin. The enterosorbent AC1 in primary beads appeared to be less effective.

In conclusion, the enterosorption session in rats with severe intoxication caused by CCl_4_ injection mitigated manifestations of oxidative stress, exerted a positive influence on the structural-morphologic indexes and regenerative potential of liver and promoted deliganding and recovery of conformation of albumin molecule. Our results could be used as a background for further investigations of carbonic enterosorbents, including the development of the third version of ImmutriX enterosorbent AC3, unifying of both types of granules in the same formulation, and their usage in therapy of acute and chronic liver failure.

## Declarations

### Author contribution statement

Veronika Sarnatskaya, Victor Mikhailenko: Conceived and designed the experiments; Performed the experiments; Analyzed and interpreted the data; Wrote the paper.

Igor Prokopenko, Larysa Yushko, Oksana Shevchuk, Alexei Glavin, Lydmila Makovetska, Larysa Sakhno, Bogdan I. Gerashchenko: Performed the experiments.

Alexei Sidorenko, Oleksandr Kozynchenko: Contributed reagents, materials, analysis tools or data.

Vladimir Nikolaev: Conceived and designed the experiments; Analyzed and interpreted the data.

### Funding statement

This work was supported by ImmutriX Therapeutics Inc., Rapid City, SD, USA. Contract V-11-17.

### Competing interest statement

The authors declare no conflict of interest.

### Additional information

No additional information is available for this paper.
